# Computed tomography–guided percutaneous biopsy in diagnosis of suspected metastatic renal cell carcinoma: which location is the most suitable?

**DOI:** 10.20452/wiitm.2024.17894

**Published:** 2024-07-31

**Authors:** Petr Hoffmann, Michal Balik, Martina Hoffmannova, Jindrich Kopecky, Pavel Ryska, Jana Draganovicova, Petr Dvorak

**Affiliations:** Department of Radiology, Faculty of Medicine and University Hospital, Charles University, Hradec Kralove, Czech Republic; Department of Urology, Faculty of Medicine and University Hospital, Charles University, Hradec Kralove, Czech Republic; Faculty of Education, Charles University, Prague, Czech Republic; Department of Oncology and Radiotherapy, Faculty of Medicine and University Hospital, Charles University, Hradec Kralove, Czech Republic

**Keywords:** biopsy location, complication rate, computed tomography guidance, metastatic disease, renal cell carcinoma

## Abstract

**INTRODUCTION:**

Systemic targeted therapy options are commonly used in patients with metastatic renal cell carcinoma (mRCC). Histological verification is crucial for treatment of mRCC.

**AIM:**

Our aim was to evaluate an optimal location for percutaneous computed tomography‑guided biopsy in a diagnosis of suspected mRCC.

**MATERIALS AND METHODS:**

A total of 138 percutaneous biopsies for tumors ranging from 21 to 133 mm in diameter (median, 72 mm) were carried out in 134 patients with suspected mRCC over a 5‑year period. The biopsy location was variable, with kidney biopsy performed in 77 cases (55.8%), and other localizations (retroperitoneum, peritoneal cavity, liver, pelvis, pleural space, lung, mediastinum, chest or abdominal wall, and pancreas) in 61 cases (44.2%).

**RESULTS:**

As many as 288 biopsies (97.1%), yielded truepositive results, and 4 procedures (2.9%) yielded histologically falsenegative results that required confirmation through extended rebiopsy. RCC was the most common individual diagnosis (85.5%), with non‑RCC histology verified in 14.5% of cases. In total, 32 complications (23.2%) were confirmed, 2 of which were pneumothoraces, 29 were minor bleeding that needed only conservative management, and 1 case required angiography and embolization for hemorrhage treatment. While no significant relationship between the biopsy success and lesion localization (renal vs other) was found (P = 0.13), the relationship between complication rate and biopsy localization (renal vs other) was significant (P = 0.01).

**CONCLUSIONS:**

Lesion localization (renal vs other) was not relevant to histological accuracy of the biop‑ sies performed in patients with suspected mRCC. However, the biopsies of lesions outside the kidney had a lower complication rate.

## INTRODUCTION

Renal cell carcinoma (RCC) is a common malignancy with an increasing incidence in the Western countries. In Europe, the highest rates are found in the Czech Republic and Lithuania.^1^^;^^2^ RCC constitutes a majority of solid renal lesions and accounts for approximately 90% of all kidney malignancies. Between 15% and 30% of newly‑diagnosed renal carcinomas have metastatic foci at the time of initial diagnosis,^1^^;^^2^ with nearly 40% of patients demonstrating metastases even after surgery.^3^ Overall survival is highly dependent on staging at the time of diagnosis; with metastatic disease having only a 12% 5‑year survival rate.^4^

For a long time, nephrectomy has been a standard therapeutic approach for patients diagnosed with metastatic RCC (mRCC), and has been widely used in clinical practice. The surgical approach was the only available treatment option due to challenges of radio and chemoresistance. However, since the introduction of targeted therapies addressing the molecular and enzymatic pathways implicated in renal cancer carcinogenesis in 2005, the treatment landscape has shifted. The intro‑ duction of inhibitors targeting vascular endothelial growth factor (VEGF), tyrosine kinase inhibitors (TKIs), mammalian target of rapamycin (mTOR) inhibitors, and the immune checkpoint inhibitors (ICIs) led to reevaluation of therapeutic strategies for disseminated diseases.^4^^;^^5^^;^^6^^;^^7^

Percutaneous biopsy was a relatively rare procedure in the era dominated by surgical interventions. Indications for nephrectomy or resection were made solely on the basis of imaging examinations, eliminating the need for biopsy and preoperative histological verification. Biopsy was reserved for cases characterized by radiologically indeterminate masses, prior to ablative treatment or in patients who were candidates for active surveillance.^8^ The role of a biopsy dramatically changed following validation of targeted mRCC therapy in clinical trials, most notably the CAR‑ MENA (Clinical Trial to Assess the Importance of Nephrectomy).^5^

Ever since, utilization of percutaneous biopsy has rapidly increased. The European Association of Urology guidelines concerning RCC encompass a comprehensive range of established principles pertaining to biopsy indications, technique, di‑ agnostic yield, accuracy, morbidity, and genetic assessment.^7^ The guidelines implicitly say that “the ideal location of core biopsies is not defined.” Consequently, the primary objective of this study was to shed some light on the problem of optimal core biopsy location.

## AIM

The study aimed to find an optimal location for computed tomography (CT)guided percutaneous biopsy in the diagnosis of suspected mRCC.

## MATERIALS AND METHODS

**Figure 1:**
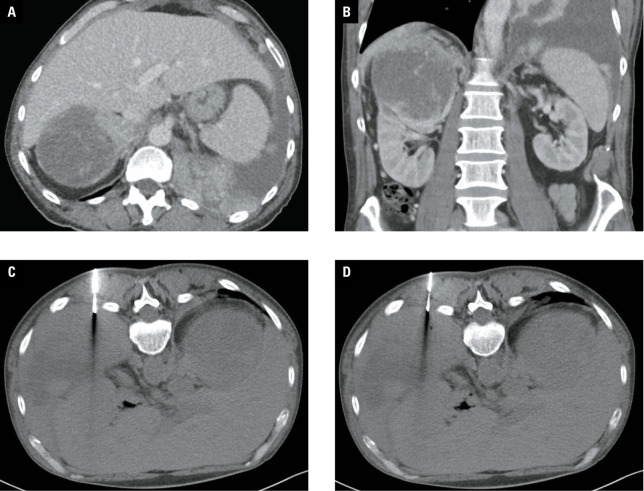
Pleural biopsy. Contrast medium-enhanced computed tomography (CT) examination (**A** – transverse plane, **B** – coronal plane) showing disseminated malignancy originating with high probability from the right kidney, with the affection of pleural space on the left side. Renal tumor with large necrotic parts. Pleural process is more viable, the biopsy was performed from this location (patient in prone position; **C** and **D**). The final histological result was grade 3 clear cell renal cell carcinoma.

Over a period of 5 years, from April 2018 to April 2023, a retrospective evaluation was carried out in a group of 134 patients. An indication for biopsy was established on the basis of imaging examinations in which disseminated malignant process, with high probability originating from the kidney, was verified. In all cases, multiphase contrast‑enhanced CT scans of the chest, abdomen, and pelvis were conducted using a contrast medium. Positron emission tomography /CT, ultrasonography (US), or magnetic resonance alone were never utilized for decision‑making but were rather employed sporadically for additional diagnostic specificity. All patients were referred by a multidisciplinary council (radiologist, oncologist, and urologist) with continued systemic therapy. The biopsy location (kidney or other organs) was selected by experienced radiologists, prioritizing safe access to tumorous tissue as the most relevant condition, with viable processes being preferred over necrotic tissues Figure 1.

Monitored parameters included age, sex, location of the lesions and the largest diameter at the biopsy level, needle gauge, number of biopsy at‑ tempts, history of previous tumorous diseases, complications and their management, final histological results including subtyping, and correlations with the outcomes of appropriate therapies.

The biopsies were performed in various anatomical locations. Table 1 provides a detailed overview. The patients were positioned supine, prone, or on their side on the CT table, with emphasis on safety and procedural accuracy. All presented interventions were performed under CT guidance using Siemens Somatom Definition AS Plus and Somatom Force equipment (Siemens, Forchheim, Germany). Neither CT fluoroscopy guidance nor any navigation system were used.

Fully informed consent was obtained from all patients by the physician performing the biopsy, with an explanation of procedural principles, potential consequences, possible complications, and their respective solutions. The study was approved by the institutional ethics review committee (202401P05).

All biopsies were performed under local anesthesia (trimecaine, Zentiva, Prague, Czech Republic); neither general anesthesia nor conscious sedation were required. Standard preprocedural assessment included examination of blood coagulation parameters, activated partial thromboplastin time (below 1.5) and international normalized ratio (below 1.5).

The intervention was planned based on diagnostic whole‑body CT examination. The needle track was carefully chosen to avoid injury to critical structures, particularly major blood vessels, bowel, or lungs, which could pose significant risks to the patients. The entry point was defined by placing a mark on the skin. The length and angle of the biopsy needle were determined using intra‑ procedural CT imaging, employing a local anesthesia needle within the patient’s body. This step was essential for accurate assessment of the needle position and localization of the lesion and the surrounding structures. The procedures were carried out using the Core halfautomatic biopsy system (Palium biopsy, M.D.L. SRL, Delebio, Italy). The anticipated needle entry site was dis‑ infected and covered with sterile drapes according to a standard protocol. Following the predetermined trajectory, a 16G/18G needle 10, 13, 16, or 20 cm in length and a throw of 22 mm was inserted into the lesion. After obtaining the specimen, the needle was carefully withdrawn from the anatomical site. The obtained samples were preserved in a sterile 4% formaldehyde solution and transported to a laboratory for further examination. A one‑step approach was employed in all cases.

After the intervention, a series of CT scans within the same range as in the preprocedural examination were performed to exclude the possibility of early complications. The needle puncture site was disinfected and covered with a sterile band‑aid. A full‑cycle intervention never exceeded 20 minutes. The patients were transported to a standard department of urology or oncology for vital sign monitoring. The following day, they were discharged following laboratory, clinical, and X‑ray or ultrasound examinations.

**Table 1:** Percutaneous biopsy location

Location	Procedures (n = 138)
Kidney	77
Other	61
Retroperitoneum	20
Peritoneal cavity	9
Liver	6
Pelvis	6
Pleural space	6
Lung	5
Mediastinum	3
Chest wall	3
Abdominal wall	2
Pancreas	1

Resection therapy was never indicated; all patients received non‑surgical treatment. Histological results were compared with known tumorous diseases in patients’ clinical history and the outcomes of the respective treatments. If, despite therapy, disease progression was con‑ firmed and the patient died, histological results of the autopsy were compared with the biopsy outcomes. An identical CT device was used for follow‑up examinations.

**Figure 2:**
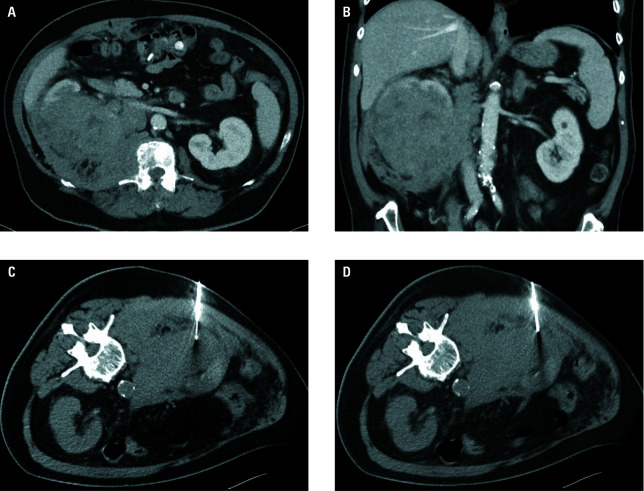
Biopsy for non-RCC process. Vascularized tumor of the right kidney and the perirenal space on contrast medium-enhanced computed tomography examination in transverse (**A**) and coronal (**B**) plane. Two percutaneous biopsy samplings were taken from different tumor parts (patient in semiprone position; **C** and **D**). The final histological result was diffuse large B-cell lymphoma.

The final histological results were supplemented with subtyping in the vast majority of cases. Decisions regarding the extent of subtyping were made by the treating urologists and (hemato)oncologists. In the cases of RCC, individual histopathological subtypes, nuclear or nucleolar grading, and the possible occurrence of sarcomatoid features were determined. In non‑Hodgkin lymphoma diagnoses, subtyping was essential; reliable differentiation between the indolent type (plasmacytoma), marginal zone lymphoma (MZL) or lymphoplasmacytic lymphoma, and aggressive types of diffuse large B‑cell lymphoma (DLBCL) determined treatment and prognosis Figure 2 In the cases of dedifferentiated carcinoma, the primary origin of the process was not identified.

### Statistical analysis 

Data for the study were collected retrospectively. Median and interquartile range were used for basic quantitative statistical evaluation. These data were then correlated with other qualitatively monitored parameters using contingency tables and the Fisher exact test. A P value below 0.05 was deemed significant. Statistical analysis was performed using NCSS 11 software (NCSS, LLC, Kaysville, Utah, United States).

**Table 2:** Histological diagnosis of the biopsied lesions

Diagnosis	Procedures (n = 138)
RCC	118
Non‑RCC	20
Urothelial carcinoma	3
DLBCL	3
Marginal zone lymphoma	2
Liposarcoma	2
Squamous cell carcinoma	2
Plasmacytoma	1
Lymphoplasmacytic lymphoma	1
SCLC	1
Dedifferentiated carcinoma	1
No tumorous cells verified (including false negative results)	4

Abbreviations: DLBCL, diffuse large B‑cell lymphoma; RCC, renal cell carcinoma; SCLC, small‑cell lung cancer

## RESULTS

A total of 138 interventional procedures with accessible final histological results were included in the study, and they were per‑ formed in 134 patients. There were 103 men (76.9%) and 31 women (23.1%), at an age ranging from 39 to 87 years (median, 68.5 years). Histological results were considered false negative in 4 cases (2.9%), prompting extended prebiopsy for histological verification. The remaining interventions (97.1%) yielded true positive results. Notably, 45 procedures (32.6%) were performed in patients with no history of malignant disease.

Histologically, the most common diagnosis was RCC, its various subtypes were identified in 118 cases (85.5%). Non‑RCC histology was identified in 20 interventions (14.5%), with lymphoma being the most prevalent condition in this subgroup. Non‑clear cell RCC (non‑ccRCC) histology was identified in 46 cases (33.3% of the interventions), a finding of significance, particularly for oncological clinical trials. No malignant cells were detected in 4 procedures (2.9%). Detailed data are presented in Table 2 and Table 3 Additionally, sarcomatoid features were determined in 15 verified cases of RCC (12.7%).

The 16G needle was utilized in 114 procedures (82.7%) and the 18G needle in 24 biopsies (17.3%). The 16G caliber was satisfactory for the majority of interventions across various anatomical sites. The use of the smaller needle caliber (18G) was reserved for high‑risk lesions located near major vessels, cases with long skin‑to‑lesion distance, and lung lesions. Statistical analysis with the Fisher exact test revealed a nonsignificant relationship between the needle gauge and both complication rate (P = 0.22) and histological accuracy (P = 0.54).

**Table 3:** Subtyping of renal cell carcinoma

Subtype	Grading	Procedures (n = 118)
Clear cell	1	8
	2	35
	3	36
	4	13
Papillary	1	2
	2	8
	3	6
	4	1
Chromophobe	3	1
	4	4
Unclassified	3	3
	4	1

The number of biopsy attempts varied across procedures, as 1 sampling was performed in 18 procedures (13%), 2 samplings in 72 interventions (57.2%), 3 samplings in 37 biopsies (26.8%), and 4 samplings in 11 cases (3%). A single attempt was determined especially for high‑risk and lung biopsies, and was often associated with the use of the 18G needle. Obtaining 2 or more samples was deemed adequate in the majority of cases (87%). Additional samplings were optimal for large lesions, with an emphasis on targeting different parts of the lesion. The Fisher exact test revealed a nonsignificant relationship between the number of biopsy attempts and histological results, including subtyping (P = 0.34). On the other hand, a significant relationship was confirmed between the number of samplings and complication rate (P = 0.001), attributed to higher incidence of complications with 3 attempts.

Percutaneous biopsies were performed at various anatomical sites for tumors ranging in diameter from 21 to 133 mm at the biopsy level (median, 72 mm). Small lesions, with the largest diameter equal to or below 30 mm, were biopsied in only 5 cases (3.7%); these lesions were located in the chest and abdominal wall. Statistical data analysis revealed a nonsignificant relationship between the lesion size, histological accuracy (P = 0.53), and complication rate (P = 0.32).

**Figure 3:**
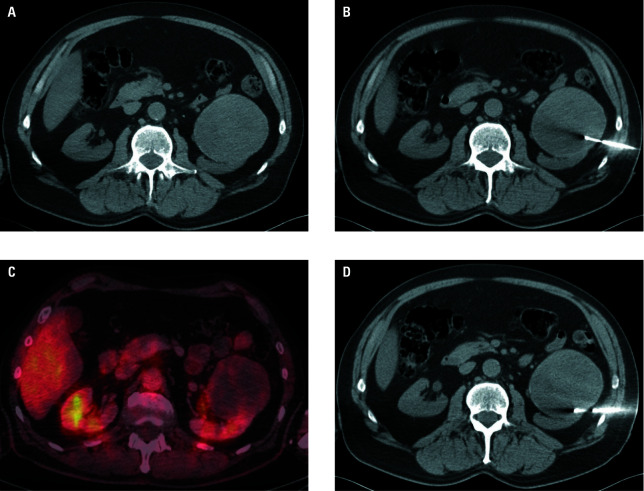
Rebiopsy. Homogenous tumor of the left kidney on nonenhanced computed tomography (CT) examination (**A**) due to transient renal dysfunction. Standard biopsy needle placement (patient in supine position; **B**). The histological result was necrotic tissue. The positron emission tomography / CT examination in transverse plane revealed viable tumorous areas on the process border (**C**) and rebiopsy needle localization (**D**). The final histological result was grade 2 papillary renal cell carcinoma.

The group with false negative final results comprised 4 patients (2.9%). In these cases, biopsy findings revealed necrotic inflammatory tissue and chronic tubulointerstitial nephritic changes. However, suspicion of malignant renal process based on imaging and clinical assessment remained high. Consequently, percutaneous prebiopsy was performed in these patients, confirming the presence of malignant histological diagnosis, including clear cell RCC in 3 individuals and papillary RCC in 1 person, each with grade specification. Although a standard biopsy technique was used, initial samples were obtained from the nonviable or central parts of the lesion Figure 3 All false negative results were confirmed through biopsy of large lesions; diagnostic success was complete in a subgroup of small foci. Surgical excision biopsy or cytoreductive resection were not indicated in the patients from our cohort.

The location of the biopsied lesions varied. The kidney was the most common location, with biopsies performed in 77 cases (55.8%). Other locations were selected in 61 cases (44.2%); in this subgroup the retroperitoneum was the most common location. Histological accuracy was compared with the needle gauge and the number of samplings, with a nonsignificant relationship. Additionally, the relationship between biopsy success and lesion localization (renal vs other) was proven nonsignificant (P = 0.13). No individual histological diagnosis correlated significantly with the final diagnostic accuracy.

The patients underwent pre‑biopsy nephrectomy in 14 cases. The time from the surgery was 33 to 156 months (median, 54 months). Biopsy involved the contralateral kidney (n = 3), retroperitoneum and liver (n = 3 each), pelvis and abdominal cavity (n = 2 each), and pancreas (n = 1). Two different histological subtypes were verified after nephrectomy in 3 patients. Histologically, identical subtypes were demonstrated in 10 cases, and different subtypes in 4 cases.

**Figure 4:**
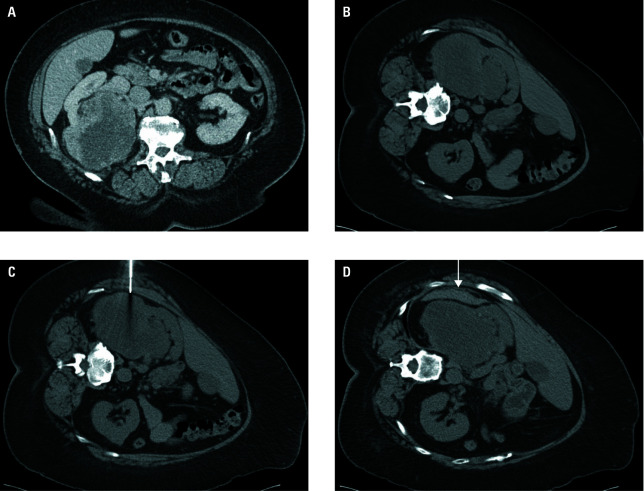
Minor bleeding. Contrast medium-enhanced computed tomography (CT) examination verified tumorous process in the right kidney (transverse plane, **A**). The biopsy was performed with the patient positioned on their left side (**B** and **C**). CT examination straight after the intervention revealed a small hematoma (arrow; **D**) surrounding the biopsied lesion. The final histological result was grade 3 clear cell renal cell carcinoma.

**Figure 5:**
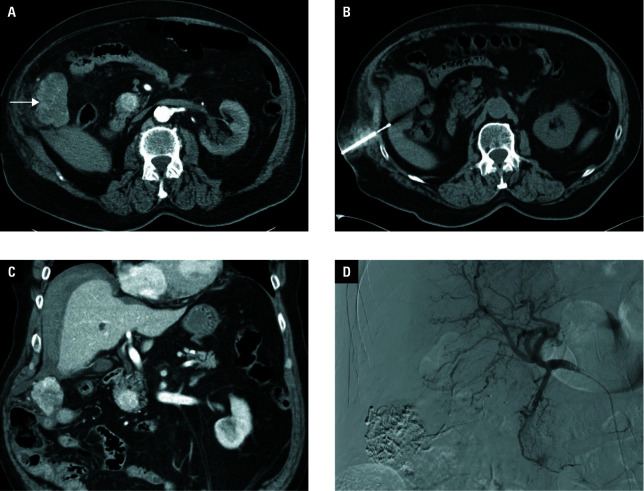
Major bleeding. Disseminated malignancy was verified based on contrast medium-enhanced computed tomography (CT) examination in transverse plane (**A**). Vascularized tumors were localized in the perihepatic space (white arrow) and in the head of the pancreas (green arrow). The placement of the biopsy needle is showed in supine position (**B**). A few hours after the procedure the patient condition worsened; CT examination was performed, revealing a large hematoma (arrow) in the perihepatic space (coronal plane, **C**). The patient underwent angiography with embolization and bleeding arrest (**D**). The final histological result was grade 4 clear cell renal cell carcinoma.

In total, 32 early complications were detected. Two procedures resulted in small pneumothoraces due to the biopsy needle penetration through the pleural line. Both patients were successfully managed with conservative treatment, with their hospital stay extended by 1 day, resulting in a total of 2 days. The remaining 30 early complications were hemorrhages, with the majority of bleeding occurring in kidney biopsies (24 cases; 75% of all complications). Two hemorrhages were observed after retroperitoneum lesion biopsy, and the other 4 hemorrhages were seen after mediastinum, pancreas, lung, and abdominal cavity biopsies. Of 30 cases of bleeding, 29 were minor hemorrhages Figure 4 A conservative approach involving observation and analgesia proved sufficient, and blood transfusion was not necessary. These patients stayed in the hospital for an additional day, making it a total of 2 days, as for pneumothorax. In 1 case, the patient objective and subjective conditions gradually worsened, and blood transfusion and angiography with embolization for bleeding arrest were necessary Figure 5 This patient was hospitalized for 7 days. All patients received follow‑up care from appropriate clinicians, addressing both complications and the extent of their disease.

In 27 cases of hemorrhage, the histological results indicated RCCs, while in 3 cases, urothelial carcinoma, dedifferentiated carcinoma, and lymph plasmatic lymphoma were identified. Statistical data analysis yielded a nonsignificant relationship between the incidence of complications and histological result (P = 0.14). However, a significant correlation was found between complication rate and biopsy localization (renal vs other; P = 0.015).

A total of 7 patients (5%) were diagnosed with lymphoma. DLBCL was confirmed as the initial diagnosis in 2 biopsies, and once as a transformation of MZL. MZL was initially diagnosed in 2 cases, while plasmacytoma and lymph plasmatic lymphoma were each confirmed once.

## DISCUSSION

We report our findings on percutaneous biopsy using CT guidance, focusing on identifying the optimal location for establishing the diagnosis of mRCC. The overall accuracy was high in renal and other (retroperitoneum, peritoneal cavity, liver, pelvis, pleural space, lung, mediastinum, chest or abdominal wall, and pancreas) biopsies. Almost 15% of the histological results were non‑RCCs. The relationship between biopsy accuracy and lesion localization (renal vs other) was insignificant, while that between the complication rate and the biopsy localization was significant, with a lower incidence of complications in the non‑kidney biopsies. A majority of the complications were minor hemorrhages.

Historically, renal tumors have been verified based on clinical examination and treated surgically. Radical nephrectomy, first described in 1948, emerged as a standard surgical approach.^9^ The procedure has undergone several changes since, particularly with the advent of laparoscopic, robotic, or nephron‑sparing approaches.^10^^;^^11^^;^^12^ The role of imaging modalities has evolved significantly. US and CT techniques, in particular, have played a fundamental role in diagnosis over the last few decades. Surgical procedures were also widely employed in the cases of disseminated renal malignancies. Cytoreductive nephrectomy was a standard of care due to chemo and radio resistance of RCC.^13^^;^^14^^;^^15^ Diagnosis and disease staging were established on the basis of imaging examinations, with surgical treatment following. Prediction models for RCC were also developed for disease‑free interval evaluation.^16^ Remarkably, nearly 20% of the resected tumors were found to have benign histology in meta‑analyses.^17^^;^^18^.

For a long time, renal mass biopsy has played a marginal role in clinical practice, and was indicated for lesions with radiologically indeterminate features, candidates for active surveillance of small tumors, or before ablative treatments.^19^ The situation changed radically after the introduction of targeted treatments (VEGF, TKIs, mTOR inhibitors, and ICIs) over the last decade. Consequently, biopsy indications expanded to include cases of suspected metastatic disease originating from RCC, often as an alternative to cytoreductive surgical procedures.

Fine‑needle aspiration biopsy and/or cytology techniques (FNAB/Cs) can sometimes be used in indicated cases, that is, if the lesion exhibits a partially fluid character. However, for precise final diagnostics, including subtyping, FNAB/Cs are not suitable.^7^^;^^19^^;^^22^ Only compact, nonfragmented, and verifiably obtained lesion samples are appropriate for histopathological examinations, therefore core needle biopsy, under only local anesthesia, is the method of choice.^7^^;^^18^^;^^19^ There is a satisfactory agreement for histological subtyping between renal tumor biopsy and surgical procedures, with reported concordance rates of around 90%.^24^ When evaluating the nuclear or nucleolar grade, a 2‑grade system (low vs high grade) demonstrates acceptable concordance at 87%, whereas a 4‑tier system (grade 1 to 4) exhibits concurrence at only 62.5%.^19^

Imaging method guidance is essential for accurate targeting of renal focal lesions. US and CT are the most commonly used modalities, with a similarly high diagnostic yield.^18^ A meta‑analysis shows that around 8% of biopsies yield nondiagnostic results.^19^ While many studies focus on small renal masses, which exhibit slightly lower diagnostic accuracy^20^ metastatic diseases typically present with large renal lesions, commonly exceeding 10 cm at the largest diameter, and containing extensive central necrotic regions. Biopsy procedures should specifically target viable tissue, that is, the peripheral tumor regions, and separate solid areas using several (at least 2) samplings.^19^^;^^21^ Our findings and experiences correspond with the existing data. The viable parts of the tumor can be preprocedurally visualized using a contrast medium, which is a standard procedure in CT‑guided biopsies; alternatively, microbubbles and US can be used.^23^ While US guidance is effective for verification of renal, liver, or abdominal wall focal lesions, its utility is limited for detecting metastatic disease affecting other organs (ie, retroperitoneum, pancreas, peritoneal cavity, pelvis, bones, mediastinum, pleural space, or lungs) due to inherent limitations of the US principles.

The overall diagnostic efficacy of CT‑guided renal tumor biopsy is similarly high to that of US navigation. Our findings (97.1% success rate) correspond to the existing data. One indisputable advantage of CT guidance is its ability to provide precise imaging records and its storage capabilities. Biopsy site selection can be demonstrated in all samplings, in addition to preprocedural imaging examinations, incorporating navigation systems with or without artificial intelligence software.^25^^;^^26^ The principal benefit of the CT approach is its ability to target focal lesions in almost all anatomical locations suspected of metastatic etiology, including the locations mentioned before.^27^^;^^28^^;^^29^^;^^30^^;^^31^ The reported diagnostic accuracy of CT‑guided biopsies in these localizations is also high. However, a substantial drawback of CT guidance is the use of ionizing radiation. Additionally, real‑time procedural monitoring is not as applicable or com ‑ mon as it is for US guidance. The use of a navigation system or CT fluoroscopy can enable real‑time intervention monitoring, especially in biopsies involving movable structures.^32^

Overall, CT guidance is the only imaging method capable of navigating to practically all affected anatomical regions in metastatic diseases, including mRCC, with high overall accuracy, even in biopsies of focal lesions outside the kidney.

Various complications have been documented from biopsies at various anatomical sites. Pain at the biopsy site is very common, but it is easily treatable. Bleeding into the retroperitoneal space or spontaneous resolution of perinephric /peritumoral hematomas have been reported in 4.3% of cases in a pooled analysis, though clinically significant bleeding is uncommon and occurs in 0.7% of cases, according to meta‑analyses.^7^^;^^19^^;^^33^ Postprocedural hemorrhage is the most frequent complication and, in sporadic cases, can be life‑threatening.^34^ The incidence of severe bleeding complications in the present study was 0.7%, with an overall complication rate of 23.2%. While our study’s findings regarding severe complications align with the existing literature, the overall complication rate was slightly elevated, though all identified complications were clinically insignificant. While needle track seeding has been described as anecdotal in large series for RCC,^35^ in the present study, tumor process seeding was not observed.

The limitations of our study include its reliance on a single‑center data collection and retrospective data analysis. Nevertheless, the number of engaged patients is statistically sufficient for the primary end point of the study.

## CONCLUSIONS

The optimal biopsy site for suspected mRCC (renal vs other) remains statistically undetermined, as both groups exhibited almost identical diagnostic accuracy. A biopsy from lesions outside the kidney returning a positive result automatically confirms disseminated malignancy. Extrarenal biopsies demonstrated a significantly lower incidence of complications, although the vast majority of verified complications were minor hemorrhages that did not re ‑ quire treatment.
